# The Book World of Medicine and Science

**Published:** 1894-02-24

**Authors:** 


### THE BOOK WORLD OF MEDICINE AND SCIENCE.

### BOOK REVIEWS.

"THUS SHALT THOU LIVE:" Hints and Advice for the Healthy and the Sick on a Simple and Rational Mode of Life, and a Natural Method of Cure. By SEBASTIAN KNEIPP, Parish Priest of Woerishofen (Bavaria), author of "My Water Cure." (London, H. Grevel and Co., 33, King Street, Covent Garden.)

Paster Kneipp's rule of life is not so original and amazing for Anglo-Saxon as it may have been for Teutonic readers. A half bath from the waist upwards every other day hardly strikes us as being a great demand on lazy human nature, and to advise farinaceous food for children and invalids instead of coffee, beer, or wine, is in these parts no new thing. One cannot help thinking that most of the worthy pastor's patients might as well have consulted their own doctors as have made a pilgrimage to Woerishofen to see and obey the clerical physician. But there they would doubtless be mpressed by the pastor's earnestness—the fervour of the man who has found it all out for himself. Apart from therepeutics, however, the pastor's opinions on things in general are not without interest. For one thing, he is convinced that manual work is the mainspring of health, and is particularly severe on girls' boarding schools (though convents do not escape his censure), because there the girls are waited upon, and, while acquiring accomplishments, forget the practical arts connected with the saucepan and the broom. As the expression of a sincere and simple soul, this book is not without interest, but as a treatise on hygiene it is somewhat gratuitous, the only point on which it differs from others being the recommendation of linen, instead of flannel, for use next the skin.

THE ELEMENTS OF HYPNOTISM: THE INDUCTION OF HYPNOSIS, ITS PHENOMENA, ITS DANGERS AND VALUE. By R. HARRY VINCENT. (London: Kegan Paul, Trench, Trübner, and Co., Limited. 1893.)

Mr. Vincent's book is superior to most of the works on hypnotism which we have met with in moderation of tone and reasonableness of claim. The protests which he utters against those who decline to have anything to do with hypnotic suggestion, and who deny its therapeutic value, are not virulent, nor does he waste his pages, as so many writers on hypnotism do, in loading them with adjectives of contempt. This is a book which all who are interested in the subject may read with interest and not without profit. But Mr. Vincent seems, like all the rest, to miss the real point of the argument against hypnotism—that it is nothing more, after all, than the power of a strong mind, accustomed to put forth its strength in a particular fashion, over a weaker mind, unpractised in such efforts. Is it not possible that an influence similar to that of the hypnotist, and more legitimate because more comprehensible and appealing to conscious, not unconscious, emotions, may be invoked by appeals to religion, patriotism, family affection, or any other of those sentiments which from time immemorial have moved mankind. Such appeals will not cure diseases, indeed, as hypnotism is said to do. But are not the diseases which hypnotism relieves rather psychical than physical in their nature? Mr. Vincent himself draws a distinction between imaginary diseases and diseases of imagination. "There is no such thing as an imaginary disease," he says. "If a man persist in thinking from Monday morning to Saturday night that he is seriously ill, the very fact that he has this impression is clear proof that either his mind or his body is diseased or out of health." Quite true, but is not such a "disease" cured as easily by any great mental shock—the death of a loved one or the inheritance of a fortune—as easily as by hypnotism? And hypnotism as yet seems to have no sure record of curing diseases of another kind than this. We would, however, commend to all Mr. Vincent's remarks on the dangers of experiments made by amateur hypnotisers, and on the necessity, for the safety of all parties, of permitting no exercise of hypnotism without the consent of the subject and the presence of reliable witnesses.

